# Harmonic Scalpel-Assisted Laparoscopic Cholecystectomy vs. Conventional Laparoscopic Cholecystectomy - A Non-randomized Control Trial

**DOI:** 10.7759/cureus.2084

**Published:** 2018-01-18

**Authors:** Kumar Rajnish, Sathasivam Sureshkumar, Manwar S Ali, Chellappa Vijayakumar, Sundaramurthi Sudharsanan, Chinnakali Palanivel

**Affiliations:** 1 Surgery, Jawaharlal Institute of Postgraduate Medical Education and Research (JIPMER), Puducherry, India.; 2 Surgery, AIIMS, Bhubaneswar, India; 3 Preventive Medicine, Jawaharlal Institute of Postgraduate Medical Education and Research (JIPMER), Puducherry, India.

**Keywords:** laparoscopic cholecystectomy, conventional cholecystectomy, electrocautery, harmonic scalpel, morbidity, operating time

## Abstract

Introduction

Laparoscopic cholecystectomy (LC) is the most commonly done, minimally invasive surgical procedure. Routinely used electrocautery produces more smoke, which masks the operating field, thereby prolongs the surgery and posing an increased risk of gallbladder (GB) perforation. The titanium clips used for clipping the cystic artery and cystic duct have a risk of slippage, which may lead to bleeding, and an increased risk for bile leakage. In addition, it may act as a nidus for stone formation. Advanced energy sources, such as the harmonic scalpel, though expensive, may provide the advantage of shorter operating time by reducing smoke, bloodless dissection in the GB bed, lower risk of bleeding from the cystic artery due to secure vessel sealing, and avoiding the use of a larger number of titanium clips. However, evidence to substantiate this advantage is limited.

Aim

To compare the operating time and perioperative complications between conventional laparoscopic cholecystectomy (CLC) and harmonic scalpel assisted laparoscopic cholecystectomy (HLC).

Methodology

All consecutive patients who underwent elective LC were included. Patients with acute infection, impaired liver function tests, concomitant common bile duct calculi, chronic liver disease/cirrhosis, suspected GB carcinoma, and pregnant women were excluded from the study. Patients were allocated into two groups. In the CLC group, both the cystic duct and the cystic artery were divided after conventional titanium clip application and electrocautery was used for thermal energy. In the HLC group, the cystic duct was clipped with a titanium clip and the rest of the procedure was carried out using Harmonic Ace (Ethicon, New Jersey, United States) and Harmonic Hook (Ethicon, New Jersey, United States). Outcome parameters analyzed were operating time in minutes, post-operative pain using visual analogue scale (VAS) scoring, frequency and route of analgesic requirement after 24 hours, and intraoperative complications, including bleeding, bile duct injury, GB perforation, and surgical site infection (SSI) in the postoperative period, per the Centers for Disease Control (CDC) criteria.

Results

Both the groups were comparable with respect to age, gender, body mass index (BMI), and the presence of comorbidity and an indication of cholecystectomy. The duration of surgery did not significantly differ between the groups (67.3 vs. 64.3 mins; p = 0.30). Other parameters, such as analgesic required on postoperative Day 1 (3.2 vs. 3; p = 0.67), VAS scores on Day 0 (4.55 vs. 4.65; p = 0.59), VAS scores on Day 1 (2.3 vs. 2.2; p = 0.84), superficial SSI (15% vs. 10%; p = 0.63), intraoperative GB perforation (30% vs. 20%; p = 0.71), and intraperitoneal drain (30% vs. 20%; p = 0.71) did not significantly differ between the groups.

Conclusion

HLC has no significant advantage over CLC with respect to operating time, postoperative pain, and perioperative complications.

## Introduction

Laparoscopic cholecystectomy (LC) is the gold standard for the treatment of gallstone disease. Conventionally, during LC, titanium clips are used for sealing the cystic duct and the cystic artery before dividing them. There have been reports of clip dislodgement, which poses an increased risk of bile leakage [1-3]. While using electrocautery, there is excess smoke production and an increased risk of lateral tissue damage. Due to the transmission of energy through the titanium clips, there is also an increased risk of gallbladder (GB) perforation. Slipped titanium clips also act as a nidus for stone formation [1,4]. The harmonic scalpel can seal vessels of up to 5 mm thickness without an increased risk of leakage [5-6].

The use of the harmonic scalpel in LC has been accepted by many surgeons. It is mainly used for the dissection of Calot’s triangle and the lifting of the GB from the liver bed. However, the cystic duct and cystic artery are divided after the application of conventional clips due to a fear of cystic artery and cystic duct leakage. The harmonic scalpel, which seals up to 5 mm thickness of luminal structures (vessels), can be used to divide both cystic duct and artery and has been shown as a safe method in a few studies [7-10]. The cystic duct diameter and thickness may vary between patients due to existing pathology, such as acute/chronic cholecystitis, fibrotic GB, etc. Hence, the ‘clipless’ LC has not been accepted widely due to the routine use of clips for the fear of cystic duct leak. Using the harmonic scalpel for the entire operative procedure of LC except cystic duct division may provide the advantage of a shorter operating time with a reduced risk of cystic duct leakage [11]. Hence, this study was carried out to assess the role of harmonic in LC (harmonic scalpel-assisted laparoscopic cholecystectomy - HLC), wherein the entire operating procedure of laparoscopic cholecystectomy was done using the harmonic scalpel, except for cystic duct division, which was done using titanium clip application, as compared to conventional LC (CLC).

## Materials and methods

This prospective, parallel arm, non-randomized controlled trial was carried out in a tertiary care center in south India over a period of two years. Institute Human Ethics Committee (IEC) approval was obtained for the study. The nature, methodology, and risks involved in the study were explained to the patient and informed consent was obtained. All the information collected was kept confidential and the patients were given full freedom to withdraw at any point during the study. All provisions of the Declaration of Helsinki were followed in this study.

The study included all patients between 18 and 70 years of age scheduled for elective LC. Patients with acute infection, impaired liver function tests, concomitant common bile duct calculi, chronic liver disease/cirrhosis, suspected GB carcinoma, and pregnant women were excluded from the study. The patients were randomly allocated to either CLC or HLC using a computer-generated random number table. Allocation concealment was carried out with the sequentially numbered, opaque, sealed envelopes (SNOSE) technique. The envelope was opened after the patients were induced under general anesthesia. The primary outcome parameter studied was operating time in minutes, which was measured starting from skin incision for port placement to the last port closure. Other outcome parameters, such as postoperative pain, analgesic requirement, rate of GB perforation, intraperitoneal drain placement, conversion to open cholecystectomy, and surgical site infection (SSI) were also compared between HLC and CLC.

Considering the reduction of 15 minutes in operating time as clinically significant, assuming a 99% confidence interval (alpha=0.01), 90% power, the sample size was calculated as 20 in each group (OpenEpi, version 3). Patients were categorized into two groups, A and B. For group A patients, the conventional four-port cholecystectomy was performed. The cystic artery and duct were clipped using titanium clips and divided. The GB was dissected from the GB fossa with monopolar diathermy.

For group B patients, the harmonic scalpel was used in the 10 mm epigastric working port for securing the cystic artery. This was done at a power setting of two and the energy source was applied continuously till the end gave way. The cystic duct was divided after securing with titanium clips. The harmonic scalpel was used for dissection throughout the procedure wherever the energy device was required. The GB was separated from the liver bed using the harmonic shear and harmonic hook. All patients received prophylactic antibiotics unless contraindicated due to hypersensitivity, renal failure etc. The duration of surgery, from skin incision to skin closure, was noted in both the groups. Postoperative pain was assessed using the visual analogue scale (VAS) score at 12 and 24 hours after surgery. At the time of discharge, during the first postoperative visit and at the 30^th ^postoperative day, patients were examined for wound infection. SSI was diagnosed according to the Centers for Disease Control (CDC) criteria. Ultrasonography (USG) of the abdomen was done during the first follow-up visit to look for any intraabdominal collection. The outcome parameters were recorded during the preoperative, intraoperative, and postoperative periods using a specified proforma.

A statistical analysis was done using the SPSS 19.0 software (IBM, Armonk, New York, United States) for Windows. All categorical data between both groups were compared using the chi-square test. Percentage of wound infections and proportions were analyzed using the chi-square test. Data related to continuous variables, such as operative time, were compared using the Independent Student t-test. Continuous variables were assessed using the unpaired t-test when they follow the normal distribution. In case of non-continuous variables as well as continuous variables not following normal distribution, appropriate statistical tests were used.

## Results

A total of 40 patients were enrolled in the study, 20 in the CLC group and 20 in the HLC group. There was no mortality in patients included in the study. The demographic profiles of the two groups were comparable. The mean age for the group undergoing conventional LC was 46.6 years ± 11.39 years, ranging from 29 years to 65 years. The mean age for the HLC group was 40.25 years ± 14.85 years, ranging from 21 years to 73 years. This suggests that cholelithiasis is most common in the age group of 40-50 years. Out of the total 40 patients enrolled in the study, 25 (62.5%) were female and 15 (37.5%) were males and the gender ratio in both the groups was comparable. Both the groups had a comparable body mass index (BMI) and distribution of various comorbidities in the study population. The American Society of Anaesthesiologist (ASA) fitness category did not significantly differ between the two groups (Table 1).

**Table 1:** Comparison of baseline parameters between the groups BMI: body mass index; SD: standard deviation; ASA: American Society of Anaesthesiologist

Parameters		Group A (n=20)	Group B (n= 20)	p- value
Age (mean±SD)		46.6±11.39	40.25±14.85	0.259
BMI (mean±SD)		23.62±4.22	23.55±4.75	0.612
Sex (n)	Male	9(45%)	6(30%)	-
	Female	11(55%)	14(70%)	-
Comorbities (n)	Diabetes	9(45%)	3(15%)	-
	Hypertension	6(30%)	4(20%)	
ASA category (n)	ASA l	11(55%)	12(60%)	-
	ASA ll	9(45%)	8(40%)	-

The mean duration of surgery in the CLC group was 67.3 ± 9.65 minutes, ranging from 54 minutes to 90 minutes (CI: 62.79-71.81). The mean duration of surgery in the HLC group was 64.3 ± 8.5 minutes, ranging from 49 minutes to 78 minutes (CI: 64.3-72.2). The difference in the duration of surgery between the two groups was not significant (p = 0.30) (Table 2).

**Table 2:** Comparison of duration of surgery (in minutes) between the groups CI: confidence interval

Surgery duration	Group A (n=20)	Group B (n=20)	p-value
Minimum	54	49	0.30
Maximum	90	78	
Mean	67.3±9.65	64.3±8.5	
Median	65	64.5	
95% CI	62.79-71.81	60.32-68.28	

The intraoperative complications between the two groups were compared (Table 3).

**Table 3:** Comparison of intraoperative complications between the groups GB: gallbladder

Variables	Group A (n=20)	Group B (n=20)	p-value
No. of patients requiring drain	6(30%)	4(20%)	0.71
GB perforation	6(30%)	4(20%)	0.71

Out of 40 patients enrolled in the study, five patients (12.5%) had intra-operative GB perforation. Three patients (15%) in the CLC group had GB perforation while two (10%) patients in the HLC group had GB perforation. The rate of GB perforation between the two groups was not statistically significant (p = 0.714). Out of 40 patients, an intraperitoneal drain was kept in seven patients (17.5%). Six patients (30%) in the CLC group and four patients (20%) in the HLC group required an intraperitoneal drain. The difference was not significant (p = 0.716).

Postoperative pain and analgesic requirement between both the groups was comparable (Table 4).

**Table 4:** Comparison of postoperative pain and analgesic requirement between the groups VAS: visual analogue scale

Variables	Group A (n=20)	Group B (n=20)	p-value
VAS day 0	4.55±0.51	4.65±0.67	0.59
VAS Day 1	2.3±0.8	2.25±0.78	0.84
Analgesic requirement (No. of diclofenac tablets)	3.1±0.8	3±0.7	0.67

All patients were given injectable analgesics in the immediate postoperative period and were given oral diclofenac tablet 50 mg from postoperative Day 1 if the patient complained of pain. An average of three diclofenac tablets was required in the HLC group while an average of 3.2 diclofenac tablets was required in the CLC group. The number of diclofenac tablets required on the first postoperative day was not statistically significant in both the groups. Visual analogue scale (VAS) on Day 0 and Day 1 of surgery for pain assessment also did not show a significant difference. The mean VAS score on Day 0 for the CLC group was 4.55 ± 0.51, ranging from 4 to 5 (CI: 4.31-4.79). The mean VAS score on Day 0 for the HLC group was 4.65 ± 0.6, ranging from 3 to 6 (CI: 4.34-4.96). The mean VAS score on Day 1 of surgery was 2.3 ± 0.8, ranging from 1 to 4 in the CLC group. The mean score of VAS on Day 1 for the HLC group was 2.25 ± 0.78, ranging from 1 to 4.

A total of five (12.5%) patients had superficial SSI. None of the patients had deep SSI. Three patients (15%) in the CLC group had superficial SSI and two patients (10%) in the HLC group had superficial SSI. The SSI rates between the two groups were not significant (p = 1.000) (Table 5).

**Table 5:** Comparison of postoperative complications between the groups SSI: surgical site infection

Variables	Group A (n=20)	Group B (n=20)	p-value
SSI	3(15%)	2(10%)	0.63
Intra-abdominal Collection	1(5%)	0	1.0

Only one patient in the CLC group had an intraabdominal collection, which was detected on USG carried out for persistent abdominal pain on postoperative Day 5. Therapeutic aspiration showed 100 ml of serous fluid. The patient was managed conservatively and the follow-up USG did not show any residual collection. None of the patients in the HLC group developed a postoperative intra-abdominal collection.

There was no incidence of major bleeding intraoperatively in both the groups and no patients in the study group required a blood transfusion. None of the patients in this study had any major postoperative complications, such as bile leakage or biliary peritonitis. None of the patients required readmission or a second surgery.

## Discussion

The safety of the harmonic scalpel as a vessel-sealing device in a high-pressure system, such as the superior, inferior mesenteric arteries and veins, has already been established. Considering the fact that the biliary tract is a low-pressure system and the thickness of the cystic duct and artery is usually less than 5 mm except in cases having previous cholecystitis/cholangitis episodes that lead to inflammatory changes, the harmonic scalpel can be safely used. The harmonic scalpel also produces less smoke and minimizes blood loss. There is also a decreased risk of GB perforation [1].  

The terminology “clipless cholecystectomy” has been used in a few studies to indicate that the total operating procedure is carried out by using the harmonic scalpel including the division of the cystic artery and cystic duct. In the present study, the harmonic scalpel was used for the dissection of Calot’s Triangle, sealing the cystic artery and the dissection of the GB from the GB fossa. The cystic duct was divided after clipping with conventional titanium clips. This was carried out to reduce the risk of bile leakage from the ligated cystic duct using the harmonic scalpel, as sealing the harmonic scalpel is limited to lumens with 5 mm thickness. Predicting the cystic duct diameter by radiological means with the help of contrast-enhanced computed tomography (CECT) also required the harmonic division of the cystic duct, which is, at times, expensive and time-consuming.

In various studies done previously, there is a significant difference in the operating time between the harmonic and electrocautery groups. This can be explained by the fact that the harmonic scalpel is a multifunctional instrument [1-2,7]. It replaces four instruments routinely used in conventional LC, namely, the dissector, clip applier, scissors, and electrosurgical hook/spatula. Hence, there is no need to change instruments repeatedly, and this saves time. Also, there is no smoke production while using the harmonic scalpel. This also saves time, as the camera lens need not be cleaned repeatedly and provides a clear operative field for the surgeon to work.

Operating time was significantly less in the HLC group in the study conducted by Jain et al. (64.7 ± 13.74 vs. 50 ± 9.36; p = 0.001) and Kadil et al. (61.88 ± 16.17 vs. 52.14 ± 9.8; p < 0.0001) [1,7]. In our study, we did not find a significant difference in operating times between the two groups (67.3 ± 9.65 vs. 64.3 ± 8.5; p = 0.30). This could be partly attributed to the use of clips for cystic duct ligation, which leads to increased operating time due to multiple instruments needed for clip application and the cutting of the cystic duct with scissors. Among the previous studies, the difference observed in most other studies, ranging from five minutes to seven minutes, has a questionable advantage when compared to the cost of harmonic scalpel [1,7,12]; only two of the previous studies had shown a reduction in operating time of 15 minutes in the harmonic group [2,13].

Bleeding during LC either from a slippage of clips from the cystic artery or bleeding from the GB fossa may make the operating field blurred, which may pose an advertent injury to the biliary system and prolongs the operating time. The safety of the harmonic scalpel for vessel ligation has been shown by Vu et al. in laparoscopic surgery [14]. As the cystic artery system is not a high-pressure zone, ligation and division of the cystic artery with a harmonic scalpel may be advantageous, as ligation and division can be done using a single instrument. Bleeding from the liver bed is also a commonly encountered problem that prolongs the operating time, as it takes time to control diffuse bleeding from the liver bed. Electrocautery with the ball tip is conventionally used to stop bleeding from the GB fossa; however, the crust formation and stickiness of electrocautery make the use of such an instrument difficult. The smoke emitted from the electrocautery also makes the operating field hazy and delays the identification of the bleeding point. A harmonic scalpel with a hook and ball dissector has the advantage of stopping the bleeding without producing smoke.

In their studies, Jain et al. and Kandil et al. have observed a significant reduction in blood loss, which was measured indirectly by means of a fall in hemoglobin and hematocrit [1,7]. No intraoperative bleeding or injury to adjacent structures was encountered in the present study. None of the patients in the present study had any fall in hemoglobin and no patients required a blood transfusion. However, a reduction in such indirect parameters for measuring intraoperative blood loss has been questioned by a few studies [13]. The clinical significance of a reduction in these parameters, which measures the few mL of additional blood loss as a statistically significant difference is also criticized by some studies [1,7]. Comparing the additional cost incurred due to the use of the harmonic scalpel, the clinically insignificant blood loss carries a questionable advantage.

GB perforation is one of the most common intraoperative complications while doing LC. The perforation also makes the operating procedure difficult and long due to the continuous leakage of bile from the perforated GB during the dissection of GB from the liver bed. Causes for GB perforation are laceration due to grasper traction and lateral tissue damage due to electrocautery dissection. The harmonic scalpel reduces the lateral thermal spread and decreases the risk of GB perforation. Kandil et al., in their study, showed that the risk of GB perforation was significantly higher in the traditional group than in the harmonic group (18.6% vs. 7.1%, respectively; p = 0.04) [7]. Conversely, the risk of GB perforation was not found significant in the study conducted by Mukesh et al. [13]. Intraoperative complications also increase the risk of GB perforation, as evident in the study of Mahabaleshwar et al., which revealed a 14.23 times greater risk of GB perforation in the presence of complications [2]. In our study, six patients (30%) in the CLC group had GB perforation while four patients (20%) in the HLC group had GB perforation; the difference was not significant with a p-value of 0.78. None of the patients in the present study had any intraoperative complications in terms of bleeding, bile duct injury, etc. The experience of surgeons and experience in handling conventional electrocautery and harmonic scalpel instruments also play a vital role in GB perforation. Careful use of electrocautery in experienced hands may lead to less incidence of GB perforation, as in the present study, where the HLC group had no significant reduction in GB perforation.

Pain in the immediate post-operative period is mostly due to visceral irritation. In the CLC group, the effect spreads laterally up to 0.5 cm as compared to 1.5 mm in ultrasonic shears. This leads to increased thermal damage and charring in surrounding tissues and nerve structures, leading to increased postoperative pain in the CLC group. Jain et al. noted that post-operative pain was significantly less in the harmonic shear group [1]. This is due to less release of inflammatory mediators, as there is less lateral tissue and nerve damage. Also, the duration of peritoneal distension is less due to the shorter surgery duration, thereby directly affecting the duration and degree of traction to vessels and nerve. Since there is less risk of GB perforation while using the harmonic scalpel, the degree of pain also reduced because of less inflammation due to bile leakage. Mahabaleshwar et al. also concluded that the postoperative pain is less in the harmonic scalpel group [2]. Post-operative pain scores after 24 hours were found to be significantly better in HCL by Kandil et al. as well (4.48 ± 1.89 vs. 3.12 ± 1.84; p = 0.000) [7]. Postoperative pain depends on the duration of surgery, personality and sensitivity of the patient, and intraoperative complications, such as biliary spillage. Since in this study, duration of surgery and GB perforation are not statistically significant, this could have contributed to the statistical insignificance of the postoperative pain score in this study.

Only a few studies compared the analgesic requirement in the CLC and HLC groups [1]. As previous studies have shown, pain scores are less in patients undergoing harmonic LC, so less analgesic is needed. But, the study conducted by Jain et al. found a significantly lower analgesic requirement in the ultrasonically activated scalpel group (2.66 ± 0.66 vs. 1.89 ± 0.59; p = 0.001) [1]. However, in the present study, no difference in the analgesic requirement was found between the two groups which could be explained by the fact that in both the groups, the VAS pain scores were similar, indicating that the postoperative pain and analgesic consumption expected to be comparable.

If bile leakage or oozing/bleeding from the vessel is suspected, intraperitoneal drainage can effectively decrease the morbidity by effectively draining out the intraabdominal contents and reducing the risk of stasis and infection. The placement of the drain increases operative time and increases morbidity. In this study, drains were kept for patients in whom significant bile spillage occurred due to GB perforation. Intraperitoneal drains were kept for six patients in the CLC group and four patients in the HLC group. The test was not significant (p-value: 0.74). This could be explained by the fact that the rate of GB perforation was not statistically significant. Also, in the study conducted by Kaul et al., no statistical difference was found in intraperitoneal drain placement in the two groups [12]. The drains in each group were removed within 48 hours.

The risk of SSI is less in laparoscopic procedures as compared to open surgeries, as the size of the incision is small. The risk of SSI depends on various factors, such as duration of surgery, spillage of bile, intra-abdominal collection due to postoperative bile leak, retrieval of GB through the port, presence of drain, and comorbidities such as diabetes. In our present study, three patients (15%) in the CLC group had developed superficial SSI. Out of three, two patients were diabetic and one patient had an intraperitoneal drain. Two patients (10%) in the harmonic group had developed superficial SSI and both patients were diabetic. The overall SSI in this study was 12.5%. In this study, the incidence of perforation of GB was comparable between the two groups and nor had we encountered complications such as bleeding or bile leakage in this study. Hence, the SSI rate of both the groups did not vary significantly with a p-value of 1.000. In all patients, infection subsided with oral cloxacillin for five days. Similar results have been shown in many studies [2,12,15].

The length of hospital stay couldn’t be assessed in the present study. Being a tertiary care and teaching hospital, the cancellation of cases were inevitable, hence, we could not assess the hospital stay of our patients. In this study, only an indirect assessment, such as a drop in postoperative hemoglobin, was carried out and an accurate quantification of intra-operative blood loss could not be assessed, which could be an important parameter. We could not randomize patients due to logistics. Further randomized trials with a bigger number of patients may provide added evidence that is not addressed in the present study.

## Conclusions

There was no significant difference in operating time, incidence of GB perforation, and intraperitoneal drain placement between the HLC and CLC groups. Further, there was no significant reduction in postoperative pain and analgesic requirements between the groups. In addition, there was no significant reduction in the postoperative SSI or intra-abdominal collection. In our study, HLC has no significant advantage over CLC. Further randomized trials are required to substantiate a clear advantage of the harmonic scalpel over conventional electrocautery for LC.
